# Pollen tube growth from multiple pollinator visits more accurately quantifies pollinator performance and plant reproduction

**DOI:** 10.1038/s41598-020-73637-5

**Published:** 2020-10-12

**Authors:** Jamie R. Stavert, Charlie Bailey, Lindsey Kirkland, Romina Rader

**Affiliations:** 1 School of Environmental and Rural Science, University of New England (UNE), Armidale, NSW Australia; 2 Department of Conservation | Te Papa Atawhai, Auckland, New Zealand

**Keywords:** Fertilization, Fruiting, Pollen, Pollen tube, Pollination, Seed development

## Abstract

Pollination services from animals are critical for both crop production and reproduction in wild plant species. Accurately measuring the relative contributions of different animal taxa to pollination service delivery is essential for identifying key pollinators. However, widely used measures of pollinator effectiveness (e.g., single visit pollen deposition) may be inaccurate where plant reproduction is strongly constrained by pollen quality. Here, we test the efficacy of single and multiple pollinator visits for measuring pollinator performance in a model plant species (apple, *Malus domestica* Borkh) that is strongly limited by pollen quality. We determined pollination success using a suite of measures (pollen deposition, pollen tube growth, fruit and seed set) from single and multiple pollinator visits. We found that pollen deposition from a single pollinator visit seldom resulted in the growth of pollen tubes capable of eliciting ovule fertilisation and never resulted in fruit or seed production. In contrast, multiple pollinator visits frequently initiated the growth of pollen tubes capable of ovule fertilisation and often led to fruit and seed production. Our findings suggest that single visit pollen deposition may provide a poor measure of pollinator performance when linked to reproductive success of plant species that are constrain by pollen quality. Alternatively, pollen tube growth from single and multiple pollinator visits can provide a measure of pollinator performance that is more closely linked to plant reproduction.

## Introduction

Pollination services from animals are critical for the reproduction of wild and cultivated plants^1,2^. Yet, accurately measuring pollinator performance in a way that captures pollinator contributions to plant reproduction is a prevailing problem^3^. With increasing concern about the loss of pollination services due to various global change drivers^4^ it is crucial that we accurately quantify contributions to pollination from different animal taxa. Specifically, we require a rigorous approach for identifying the most effective pollinators of wild and cultivated plants so that management of natural and production systems is optimised for conserving or enhancing key pollinator populations.

Quantifying pollinator performance is invariably challenging because of the complex interrelated processes involved in the transfer of pollen from donor to recipient flowers, and the processes that subsequently determine seed production^3^. This complexity has resulted in researchers employing a myriad of approaches to measure pollinator performance to estimate contributions to plant reproduction. In particular, visitation frequency is commonly used as a proxy for pollen deposition, and single visit pollen deposition on a virgin flower is regularly used as a proxy for fruit and/or seed production. Indeed, previous studies have shown that visitation frequency is correlated with plant reproduction^5–7^. However, such proxy measures may not accurately reflect pollinator contributions to plant reproduction because they rely on various assumptions (e.g., that all pollinators are equally effective or that all pollen deposited is capable of eliciting seed production)^3^. For example, in plant species that are self-incompatible, multi-flowered or have highly specialised reproductive structures, such assumptions are unlikely to hold, resulting in inaccurate estimates of pollinator performance^8–10^. Similarly, seed production from single pollinator visits is frequently used to measure pollinator performance, but is strongly influenced by interactions with external conditions that are not directly related to pollination (e.g., availability of maternal resources, water, nutrients and light)^11^. This may result in estimates that are not necessarily reflective of pollinator performance for the purpose of understanding pollination success.

Reproduction in animal-pollinated plants is constrained by the quantity and quality of pollen deposited by pollinators^12^. To fertilise an ovule and sire seeds, pollen grains must be conspecific^13,14^, viable^15^ and compatible^16,17^. Although pollen quantity and quality both constrain plant reproduction additively, pollen quantity is only important at the lowest range of pollen receipt. Once a sufficient number of pollen grains are deposited for full fertilisation, pollen quality becomes the primary driver of pollination success^12^. Thus, for self-incompatible plants, the qualitative component of pollination is far more important than the quantitative component because the deposition of self-pollen, regardless of its quantity, will not result in ovule fertilisation. Accordingly, pollen quality limitation is likely a chronic problem for many plant species but is disregarded by studies that simply measure pollen deposition on the stigma to quantify pollinator performance.

Identifying whether pollen is conspecific and viable is relatively straightforward^18^, but determining pollen compatibility is highly complex and depends on the plant breeding system, degree of self-compatibility and previous behaviour of the pollinator. Although genetic markers could provide a direct measure of outcrossed versus self-pollen delivered, such methods present substantial time and exorbitant financial costs to researchers^10^. Alternatively, measuring pollen tube growth from single or multiple pollinator visits provides an accurate measure of both pollen quality and quantity. In many self-incompatible plant species, tube growth of self-pollen is inhibited on the stigma or in the style, so only pollen grains from compatible donors will form pollen tubes capable of reaching the ovule(s) to potentially sire seeds^19,20^. Thus, by accounting for both pollen quantity and quality, measuring pollen tube growth rather than stigmatic pollen deposition may provide a more meaningful measure of pollinator performance that is linked to plant reproduction.

Here, we assess different measures of pollinator performance in a model plant species that is reproductively constrained by both pollen quantity and quality. Specifically, we focus on pollination of a self-incompatible apple variety (*Malus domestica* Borkh “Pink Lady”) by honeybees (*Apis mellifera*). Pink Lady apple flowers require the delivery of viable out-crossed pollen to reproduce^21,22^, while honeybees are ubiquitous and effective pollinators for most food and fibre crops worldwide^1,6^. Thus, we focused on four research questions: (1) Can a single pollinator visit deposit enough compatible pollen to produce pollen tubes capable of eliciting plant reproduction? (2) Do pollen deposition and pollen tube growth increase with successive pollinator visits and if so, is the degree of increase equal for all pollen tube development stages? (3) Do more pollinator visits per flower equate to higher plant reproductive success?

## Methods

### Study sites

We carried out our pollination experiments in September (austral spring) in 2017 and 2018, on 12 orchards near Stanthorpe, Queensland, Australia. Apple growers in this region follow a standard conventional management style. Apple trees are grown in rows, approximately 4.5 m apart, with trees planted approximately every 1.25 m within rows. Orchards used in our study ranged between 41,000 m^2^ and 104,000 m^2^ in size. In this region, “Pink Lady” and “Gala” are the most common apple cultivars.

### Apple floral biology

We used the “Pink Lady” cultivar of apple, *Malus domestica*, as a model pollination system because it requires outcrossed pollen delivered by a pollinator vector to produce fruit and seed^21,22^. Apple is monoecious and has a floral morphology typical of the Rosaceae family^23^. The flowers are hermaphroditic and grow in cymes of five, with the apical flower being the most advanced. Each flower typically consists of five petals, 20–25 stamens and five stigmas that unite into a common style which leads to the ovary. The ovary has five carpels that each contain two ovules (i.e., each flower can produce a maximum of ten seeds).

### Single and multiple visit pollination trials

To record single and multiple pollinator visits, we first covered inflorescences in the late balloon stage (stage E; Fig. S1) with organza bags to prevent pollinator visitation. If inflorescences had open flowers, we manually removed them to prevent pollen contamination within the bagged inflorescence. To further prevent pollen contamination, the bags were clipped to the stem below the inflorescence and the top was billowed out around the developing flowers to prevent the flowers from rubbing against the fabric. All selected flowers were from trees in rows containing only the “Pink Lady” cultivar and were within orchard blocks that had two to five apple varieties.

Directly following anthesis (1–2 days after flowers were bagged), we exposed flowers to one of four treatments: (*i*) hand pollination with a compatible polliniser (hand-pollinated), (*ii*) flowers left open to all pollinators for 48 h (open-pollinated) so that any number of pollinator visits could be received, (*iii*) pollinator exclusion (control), and (*iv*) pollinated by one to twelve visits by honeybees, *Apis mellifera*, (visited). On all orchards in our study and throughout most of Australia, *Apis mellifera* is an introduced species and exists both in wild colonies that live in tree hollows and other cavities, and in colonies managed by beekeepers. We only recorded visits where honeybees contacted at least one of the five stigmas on the open flower. Rapid visits (< 1 s) were included, providing the honeybee’s body visibly contacted a stigma. Once the desired number of visits had occurred, we removed stamens from flowers using fine tipped forceps to prevent further self-pollen contamination. Any closed flowers were removed from the inflorescence to prevent pollen contamination to the visited flower. We then re-covered flowers with an organza bag to prevent any further pollinator visits until the stigmas were collected 48 h later. We used un-visited open flowers as non-pollinated controls.

To hand pollinate flowers, we first collected donor flowers from apple varieties other than “Pink Lady” and stored them in chilled coolers (mean temperature = 22.6 °C, minimum temperature = 18.3 °C) within 50 mL plastic vials^24,25^. Donor flowers were collected from co-flowering apple varieties present within the corresponding orchard block, which reflected the pollen available to pollinators. We removed donor flower anthers from their filaments with fine forceps and left them in an open vial, at room temperature (14–25 °C), for 24 h to stimulate anther dehiscence. We then transferred anthers into a microcentrifuge tube and used a paintbrush to deposit pollen onto the stigmas of recipient flowers. The paintbrush was dipped into the dehisced pollen once per pollination event so that pollen deposition effort was equal across replicates. In each pollination event, all stigmas on flowers were contacted and therefore potentially received some pollen. We only used pollen for hand pollination that had been collected within 24 h.

### Measuring pollen deposition and pollen tube growth

Protocols for preparing gynoecia to view pollen tubes vary widely, even for the same plant species^26–28^. We followed existing general protocols to prepare gynoecia for microscopy including methods to fix, soften, stain and squash styles^29^. However, we optimised pollen tube visualisation methods for Pink Lady apple styles by modifying several steps as follows. Firstly, we removed styles from flowers 48 h after pollination and fixed them in absolute ethanol. Following fixation, we softened styles with 3 M NaOH for 24 h, and then submerged them in distilled water for an additional 24 h to remove the NaOH solution. After this we performed a two-step staining process. First, we added 0.1%_aq_ Fuchsin Red solution to the distilled water to stain the pollen grains. Then, we squashed and mounted styles on microscope slides in a drop of decolorized 0.1% aniline blue buffered with 0.1% K3HPO4, and let it stand for 4 h to stain the pollen tubes^30^.

To count the number of pollen grains deposited on each stigma, we took digital photographs of stigmas with a Nikon Eclipse 90i microscope in bright-field mode. We then counted the total number of pollen grains on each stigma using the “count tool” in Adobe Photoshop v 10.0. To count the number of pollen tubes at each location down the style, we observed three stylar regions with the Nikon Eclipse 90i microscope in fluorescence mode. “Stylar region one” was the region directly below the stigma, “stylar region three” was the region at the bottom of the style, and “stylar region two” was equidistant from “stylar region one” and “stylar region three”. To prevent biases when counting pollen grains and pollen tubes, we randomised samples so that the treatment group was unknown during counting. We achieved randomisation using the “RAND” function in Apache OpenOffice Calc to generate a column of random numbers adjacent to the column of sample identities. We then sorted both columns by the random number column (in ascending order) to randomise the order of sample identity rows. Then, in a new column next to the sample identity column, we assigned ascending values to each cell. We copied these new, randomised sample identities to a new spreadsheet where we then recorded the data for each sample.

### Measuring fruit and seed set

To measure fruit set (the production of fruit from flowers subjected to one of our treatments), we harvested fruit from marked flowers approximately 30 days (initial fruit set) and 6–7 months (final fruit set) after pollination treatments. We performed the initial fruit set measurement to account for pollination without the influence of fruit thinning by growers and loss due to damage caused by tractors, birds, and insect pests. To measure seed set, apples were first halved and then further dissected and cored to count the number of seeds and empty carpels. We assessed seed quality with the help of local agronomists from Orchard Services, Stanthorpe, Queensland, and a very small number of seeds (*N* = 5) were excluded as they were deemed by agronomists to be undeveloped or deformed. Flowers used to measure fruit and seed set were not the same as those used for testing pollen deposition and pollen tube growth because the effects of removing styles prior to fruit development are unknown and likely to inhibit plant reproduction. Organza bags were removed from flowers 7–14 days after pollination treatments to ensure fruit resulting from each treatment developed under equivalent environmental conditions.

### Statistical analyses

To test if the number of pollen grains on the stigma or pollen tubes at each stylar region differed between open-pollinated, pollinator excluded, hand-pollinated and visited flowers, we fitted a generalised linear mixed-effects model (GLMM) with a zero-inflated negative binomial distribution using the *glmmTMB* package^31^. The negative binomial error structure allowed us to account for overdispersion in the data, while the zero-inflation component allowed us to account for the large number of zero count observations. In this model, the number of pollen grains or pollen tubes per style was the response variable, and pollination treatment (categorical), pollen development stage (categorical), and the interaction between pollination treatment and pollen development stage were fixed effects. We included flower identity and stigma number nested within flower identity as random effects to account for our dependent data structure. We tested for differences in the number of pollen grains or pollen tubes between treatments, within each pollen development stage, using pairwise comparisons in the *emmeans* package^32^ and determined significance using FRD corrected *P* values (at *α* = 0.05)^33^. Next, to test the effect of the number of pollinator visits on pollen deposition and pollen tube growth, we fitted a GLMM as described above. However, in this model, the number of visits (continuous), pollen development stage (categorical), and the interaction between the number of visits and the pollen development stage were the fixed effects. We tested whether the slopes for pollen grains/tubes with increasing pollinator visits within each pollen development stage were different from zero, and whether there were significant differences between slopes, using the *emmeans* package.

To determine if there were differences in the probability of fruit production among our different pollination treatments, we fitted a GLMM with a binomial error distribution. In this model, the presence or absence of a fruit was the response and pollination treatment (categorical) was the fixed effect. We included flower identity as a random effect. To test for differences in seed production among our different pollination treatments, we fitted a GLMM with a zero-inflated negative binomial error distribution, where the number of seeds per flower was the response and pollination treatment (categorical) was the fixed effect. We included flower identity as random effect. Next, we tested for differences between treatments in the probability of fruit production and the number of seeds produced per flower using pairwise comparisons in the *emmeans* package. Specifically, to determine if the probability of fruit production and the number of seeds produced per flower increased with the number of pollinator visits we fitted GLMMs as specified above. However, these models were fitted to a subset of the data that included only observations from the single and multiple pollinator visit treatments. Further, in these models, the fixed effect was the number of pollinator visits (continuous). We performed model diagnostics and validated the fit of all models with the *DHARMa* package, which uses a simulation-based approach to create readily interpretable scaled residuals from GLMMs^34^.

Finally, we determined the effect of pollination treatment on pollen grain and pollen tube survival, across the different pollen development stages, with a Cox’s proportional hazards model using the *survival* package^35^. In this model, survival represents the probability of a pollen grain or pollen tube progressing to the next pollen development stage. We compared pollen grain and pollen tube survival between pollination treatments, as estimated by the Cox’s model, using pairwise comparisons in the *emmeans* package and determined significance using FRD corrected *P* values (at *α* = 0.05). We extracted pollen grain and pollen tube survival rate predictions for each pollen development stage from the model using the *survival* package. Finally, to determine whether the probability of pollen grain survival was affected by the number of pollinator visits, we fitted a second Cox’s proportional hazards model with the data as described above but specified the number of visits as continuous. We performed all statistical analyses in R version 3.5.1^36^.

## Results

**Figure 1:**
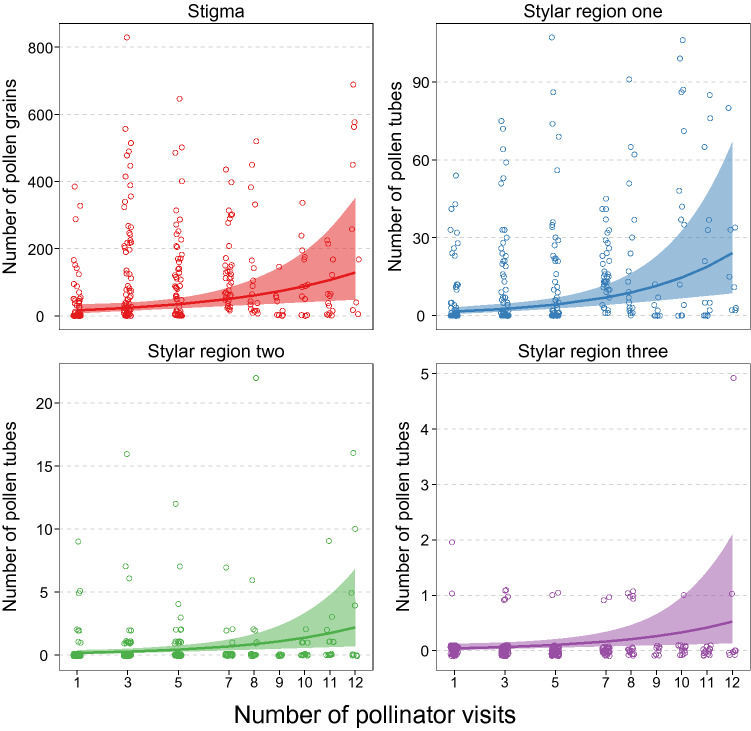
The number of pollen grains deposited per stigma and the number of pollen tubes at the top, middle and bottom of styles with an increasing number of pollinator visits. Open circles are pollen grain or pollen tube counts from individual stigmas. Solid lines are model estimates for each pollen development stage and shaded ribbons are the model estimated 95% confidence intervals.

**Figure 2:**
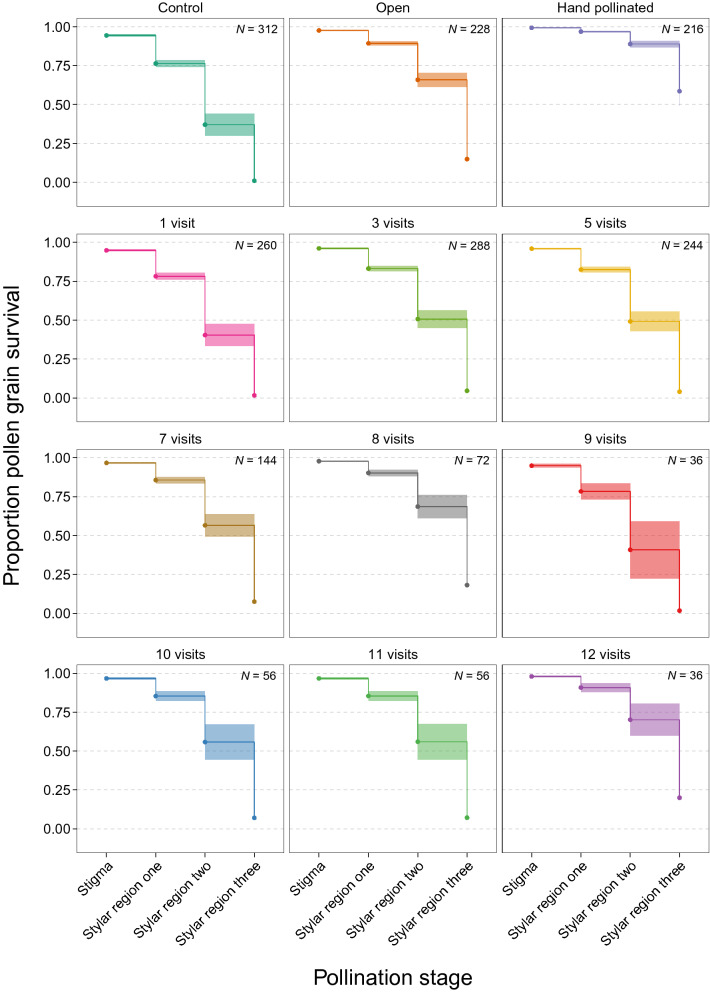
Estimates from the Cox’s proportional hazards model showing pollen grain survival probabilities across the different stages of pollination for each pollination treatment (hand-pollinated: pollinated by hand with a compatible polliniser, open-pollinated: flowers left open to all pollinators until stigmas were no longer receptive, control: all pollinators excluded, visited: flowers visited one to twelve times by pollinators). Shaded bands between each pollen development stage are the model estimated 95% confidence intervals. Refer to Table S1 for pairwise comparisons between each pollination treatment.

We found that flowers subjected to more pollinator visits received a greater number of pollen grains and subsequently developed more pollen tubes (Fig. 1). For each added pollinator visit, the chance of a pollen grain or pollen tube progressing to the next pollen development stage increased by 6% (cox model estimate ± SE = − 0.058 ± 0.013, *z* = − 4.53, *P* < 0.0001; Fig. 2). For example, after one pollinator visit, stigmas had a 1.6% chance of growing pollen tubes to the bottom of the style, whereas stigmas that received 12 visits had a 20% chance of growing pollen tubes to the bottom of the style. Further, the slopes of the positive associations between the number of pollinator visits and pollen deposition/pollen tube growth were similar across the different pollen development stages (Fig. 1). However, this positive association was stronger for pollen tube growth at the top of the style compared to pollen deposition (Table S2).

Our fruit set results also show that increased pollinator visitation increased reproductive success (Fig. S2). Specifically, the proportion of flowers that produced fruit (model estimate ± SE = 0.39 ± 0.11, *z* = 3.70, *P* < 0.001) and the number of seeds produced per flower (model estimate ± SE = 0.22 ± 0.11, *z* = 2.09, *P* = 0.037) increased with the number of pollinator visits. Importantly, we found that single visits were insufficient for initiating plant reproduction and flowers required at least two pollinator visits to produce a fruit.

We found clear evidence of pollen quality limitation; on average, open stigmas received twice as many pollen grains (model estimate ± SE = 154.01 ± 77.20) compared to hand pollinated stigmas (model estimate ± SE = 75.65 ± 39.68), but pollen grains deposited on hand-pollinated stigmas were four times more likely to grow to the bottom of the style (Fig. S3). Furthermore, hand-pollinated stigmas received, on average, twice the number of pollen grains (model estimate ± SE = 34.17 ± 7.55) compared to stigmas exposed to limited visitation (1–12 pollinator visits), but were up to 37 times more likely to grow pollen tubes to the bottom of the style (Fig. S3). From the sigmas that received one pollinator visit, 42% developed pollen tubes but only 3% grew pollen tubes to the bottom of the style (Fig. 1).

**Figure 3:**
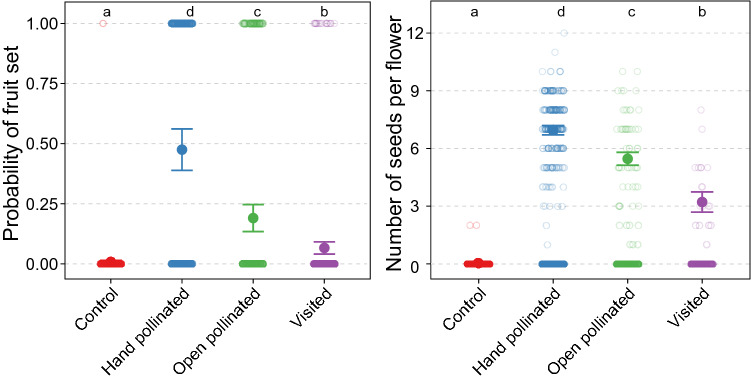
Differences in the probability of fruit production and the number of seeds produced per flower across the different pollination treatments (hand-pollinated: pollinated by hand with a compatible polliniser, open-pollinated: flowers left open to all pollinators until stigmas were no longer receptive, control: all pollinators excluded, visited: flowers visited one to twelve times by pollinators). Small semi-transparent open circles represent the raw data (presence or absence of a fruit, or the number of seeds produced per flower). Large solid circles are the model estimates for each treatment and error bars are the model estimated 95% confidence intervals. Letters above treatments denote statistically significant differences (*α* = 0.05; FDR corrected) between treatments in either the proportion of flowers that produced a fruit, or the number of seeds produced per flower.

Pollen quality limitation and the subsequent lack of pollination success was further reflected in our plant reproduction results (Fig. 3). For example, hand-pollinated flowers were seven times more likely to produce fruit compared to flowers with limited visitation (1–12 pollinator visits) and were almost three times more likely to produce fruit than open-pollinated flowers. Our seed production data also indicated pollen quality limitation wherein hand-pollinated flowers produced 74% more seed compared to visited flowers (1–12 pollinator visits) and 24% more seed than open-pollinated flowers. Finally, we found that no flowers subjected to a single pollinator visit produced fruit and seed, although even after 10 visits only 20% of flowers produced seed and fruit reflecting the pollen quality limitation in our system (Fig. S2).

## Discussion

Pollinator performance is commonly quantified using the number of pollen grains deposited on the stigma from a single floral visit^3^. However, here we show that where plants are strongly limited by pollen quality, single visit pollen deposition may not accurately capture variation in pollinator performance. This is likely because sufficient viable and compatible pollen grains capable of eliciting fertilisation were rarely deposited in single visits. Thus, where plant reproduction is strongly constrained by pollen quality, our results suggest that quantifying pollen tube growth from multiple visits can provide a more accurate measure of pollinator performance.

As we have demonstrated, pollen deposition from single or even multiple flower visits may not accurately reflect pollinator performance in terms of contribution to plant reproduction. In monoculture cropping environments, pollen quality can strongly limit fruit and seed production, given the high densities of conspecific crops and/or crop varieties. In these systems, the predominant transfer of self-pollen or pollen from genetically related donor plants is probably the rule rather than the exception. High amounts of self-pollen transfer is a particular problem for plants that require pollen from different cultivars to produce fruit and seed^37^. For example, in apple, self-pollen tube growth is inhibited in the top third of the style by proteins formed when the *S*-allele of the style and pollen tubes are recognised as equal^38,39^. Further selection then occurs as pollen tubes progress down the style, so that only the fastest growing compatible pollen tubes reach the ovules^19,20^. Like apple, in many other self-incompatible plants, the qualitative component of pollination is generally more important than the quantitative component because the deposition of self-pollen, regardless of its quantity, will not result in ovule fertilisation. However, even plant species with a high rate of autonomous self-pollination can suffer inbreeding depression if pollinators fail to deliver outcrossed pollen^40,41^. Self-pollen can also interfere with the performance of cross-pollen^17,42^ and self-pollen tubes can disable ovules in species with late-acting self-incompatibility mechanisms^43,44^. Considering that at least 24 plant families exhibit self-incompatibility to some degree, our findings are likely applicable to a wide range of plant species^44^. Thus, incorporating the quality component of pollination, especially for self-incompatible species, is critical if researchers are aiming to measure pollinator performance in a way that is relevant to plant reproductive success.

Despite the importance of measuring pollen quality in addition to quantity, determining pollen compatibility is complex due to dependency on the plant’s breeding system, degree of self-compatibility and previous activity of the pollinator^3^. While seed or fruit production resulting from single or multiple pollinator visits could provide a more direct measure of pollinator contribution to plant reproduction, there are many factors that occur between the stages of pollen tube development and fruit set which are unrelated to pollination (e.g., availability of maternal resources, water, nutrients and light)^45,46^. Thus, we suggest that quantification of pollen tubes reaching the bottom of the style may be the most representative measure of pollinator performance, as it accounts for pollen quantity and many of the factors constraining quality, while minimising the influence of variables external to the pollination process. Pollen tube quantification is also relatively time efficient, which is especially important for studies with multiple pollinator and/or plant species. However, such a method may present significant challenges for assessing performance of less common pollinator species, where quantifying pollination effectiveness for multiple animal species is the aim.

Here, we have shown that pollen deposition on the stigma provides a poor measure of pollinator performance in terms of plant reproduction (fruit and seed production). However, we focus on a single pollinator species (the European honeybee, *Apis mellifera*). Given different pollinator species are likely to affect pollen quantity and quality limitation depending on their behaviour and delivery of self-pollen and/or pollen from related plants in genetically structured populations^12^, future studies would benefit from trialling a greater number of pollinator taxa. Species with long-flight distances or erratic foraging patterns may improve the quality of deposited pollen due to higher transfer rates of out-crossed pollen^47^. For example, solitary bees are often more effective pollinators of self-incompatible plants, such as apple^48,49,^ due to their random foraging behaviour which increases the chances of pollen deposition from other varieties. Furthermore, solitary bees often carry greater amounts of cross pollen compared to honeybees, despite both taxa carrying similar total pollen quantities. Foraging behaviour can vary widely even within an animal species leading to substantial intraspecific differences in pollination performance. For example, honeybees foraging for pollen tend to deposit higher ratios of viable pollen compared to honeybees foraging for nectar^48,50^. This is because pollen-collecting individuals are attracted to young flowers with fresh pollen, whereas bees collecting nectar prefer older flowers that provide nectar more readily but have more unviable pollen^51^.

Finally, a multiple pollinator visit approach may be particularly useful for plant species in which the deposition of viable outcross pollen is low (e.g., poor pollen quality), resulting in a small probability of flowers receiving pollen capable of eliciting fertilisation. Indeed, the highest number of pollinator visits that we trialled (twelve visits) produced the best pollen tube survival rate and yielded pollen tubes that progressed to the bottom of the style. In contrast, pollen tube survival rate resulting from single pollinator visits was the lowest of all our treatments and no pollen tubes grew to the bottom of the style. Further, the multiple visit approach is a powerful tool for researchers interested in comparing fruit and/or seed production as a result of floral visitation from different pollinator taxa. Multiple visits account for the non-additive nature of pollination; specifically, pollination success is not simply the sum of all pollen grains deposited but is also dependent on subsequent pollinator interactions upon visitation (e.g., removal of pollen grains from the stigmatic surface). Indeed, our results demonstrate non-additivity wherein pollen tube growth increased more rapidly than pollen deposition with an increasing number of pollinator visits. The number of pollinator visits required to produce fruit and seed is likely to vary widely across plant species, depending on the plant’s breeding system and surrounding environment (e.g., orchard configuration, degree of habitat fragmentation, plant population genetic structure). However, flowers will likely require fewer visits where pollen quality (proportion of viable and compatible pollen) is higher, as demonstrated by our hand cross-pollination treatment. A multiple visit approach may be particularly useful for plant species with multiple ovules per flower (e.g., apple, cranberry), which require a minimum threshold number of pollen grains to elicit fruit and seed development^11,17^. In such species, plants will abort fruit even where only one carpel is left unfertilised. Therefore, studies are required across multiple plant species with different reproductive traits (e.g., degree of self-compatibility, autonomous pollination capability) to determine the point that fruit and seed production saturates with increasing pollinator visitation. Such an approach could ultimately allow meaningful comparisons of contributions to fruit and seed production among pollinator taxa.

## Conclusions

Animal-mediated pollination is a complex process that varies greatly across plant species, ecosystems, and environments. Importantly, we show that floral visitors which deposit many pollen grains do not necessarily deposit quality pollen capable of eliciting fertilisation. Hence, studies that simply measure the quantity of pollen transferred by flower visitors in a single visit may fail to capture pollination services of relevance to plant reproduction and crop production. We now require research that focuses on the effectiveness of different pollinator and plant species that vary in their reproductive traits. This will allow us to elucidate the interaction between pollinator visitation, pollination success and plant reproduction.

## Supplementary information

Supplementary Information.

## Data Availability

Data used in analyses are available at:10.6084/m9.figshare.13023224.v1.
