# Supplementary material for: Pollen tube growth from multiple pollinator visits more accurately quantifies pollinator performance and plant reproduction
Source: Sci Rep. 2020 Oct 12;10:16958. doi: 10.1038/s41598-020-73637-5 (PMC7552416; doi:10.1038/s41598-020-73637-5)
Supplement: Supplementary file 1 — Supplementary Information. [file 41598_2020_73637_MOESM1_ESM.pdf]

Supplementary Information:

Pollen tube growth from multiple pollinator visits more accurately quantifies pollinator performance and plant reproduction

Jamie R. Stavert[1,2*], Charlie Bailey[1], Lindsey Kirkland[1] and Romina Rader[1]

[1]School of Environmental and Rural Science, University of New England (UNE), Armidale, NSW, Australia.

[2]Department of Conservation - Te Papa Atawhai, Auckland, New Zealand

***Corresponding author contact:**

Email: jamie.stavert@gmail.com

Table S1. Estimatesand pairwise comparisons for the cox proportional hazards model comparing pollen grain and pollen tube survival between pollination treatments. Treatments that share letters in the "Group" column have pollen grain/tube survival rates that are not statistically different, as determined by FRD corrected pairwise comparisons (at α = 0.05).

**Treatment**	**Estimate**	**SE**	**Group**
C	0.58	0.06	f
HP	-1.55	0.16	a
O	-0.29	0.10	b
V1	0.48	0.07	ef
V3	0.20	0.08	cd
V5	0.24	0.08	de
V7	0.02	0.12	bcd
V8	-0.40	0.19	b
V9	0.47	0.20	def
V10	0.05	0.19	bcde
V11	0.04	0.19	bcde
V12	-0.45	0.29	bc

Table S2. Wald tests assessing whether the model estimated slopes for pollen deposition and pollen tube growth with an increasing number of pollinator visits are different from zero. All *P*-values are FRD corrected (at α = 0.05).

**Pollen development stage**	**Estimate**	**SE**	***t*-value**	***P*-value**
Stigma	0.19	0.07	2.80	0.0068
Stylar region 1	0.25	0.07	3.61	0.0013
Stylar region 2	0.24	0.08	3.01	0.0053
Stylar region 3	0.23	0.10	2.36	0.0186

Table S3. Pairwise contrasts between the model estimated slopes for pollen deposition and pollen tube growth with an increasing number of pollinator visits. All *P*-values are FRD corrected (at $\alpha = 0.05$).

**Pollen development stage contrast**	**Estimate**	**SE**	***t*-value**	***P*-value**
Stigma - Stylar region 1	-0.06	0.02	-3.44	0.0036
Stigma - Stylar region 2	-0.05	0.04	-1.15	0.7523
Stigma - Stylar region 3	-0.04	0.07	-0.59	0.9625
Stylar region 1 - Stylar region 2	0.01	0.04	0.31	0.9625
Stylar region 1 - Stylar region 3	0.02	0.07	0.24	0.9625
Stylar region 2 - Stylar region 3	0.00	0.08	0.05	0.9625

Table S4: Proportion of visits from different insect taxa to Pink Lady apple flowers in the orchards used for this study.

**Taxon**	**Proportion of total visits**
Honeybees (*Apis mellifera*: Hymenoptera)	92.0%
Hover flies (Syrphidae: Diptera)	2.8%
True bugs (Hemiptera)	1.4%
Beetles (Coleoptera)	1.1%
Non-syrphid flies (Diptera)	0.9%
Wasps (Hymenoptera)	0.6%
Ants (Formicidae: Hymenoptera)	0.4%
Moths (Lepidoptera)	0.3%
Wild bees (Hymenoptera)	0.3%
Butterflies (Lepidoptera)	0.2%

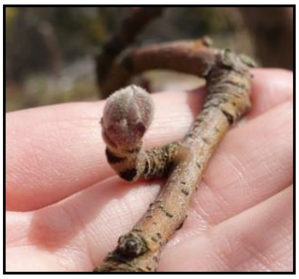

STAGE A - Dormant Bud

Brown in colour. Small, no obvious signs of green.

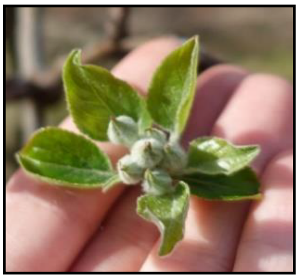

STAGE B - Green Tip AKA "spur burst"

Transition from brown to green, larger in size than dormant bud. Closed green sepals. No pink balloon visible.

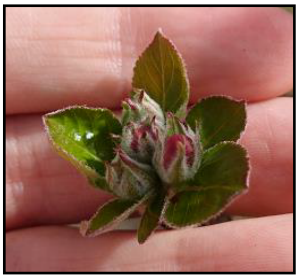

STAGE C - Pink Tip

Green sepals starting to open. Dark pink balloons slightly visible between sepals.

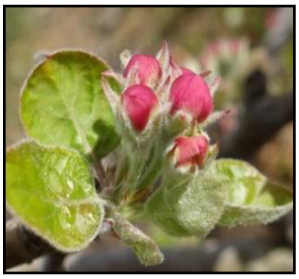

STAGE D - Early Balloon

Flowers still closed. Visible dark pink balloons between sepals. Green sepals open further.

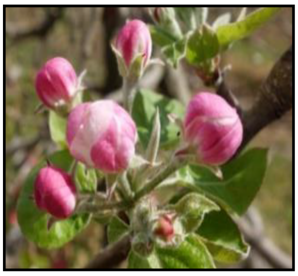

STAGE E - Late Balloon

Flowers starting to open. Petal colour transitions to pink-white as emerging. Sepals fully open. Anthers and stigma still enclosed.

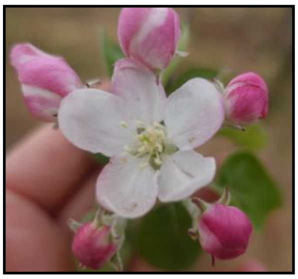

STAGE F - Open King Flower

Flower open. Anthers and stigma visible. Yellow anthers are un-dehisced to dehisced.

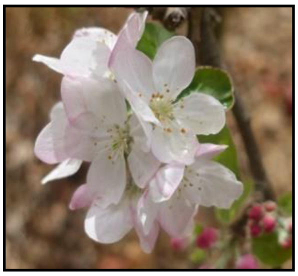

STAGE G - Open Cluster

Yellow-orange anthers. May have brown stigma tip. Cluster may stay open for 3-4 days (can be up to 7-10 depending on weather).

Figure S1. Series of images depicting the developmental stages of apple flowers.

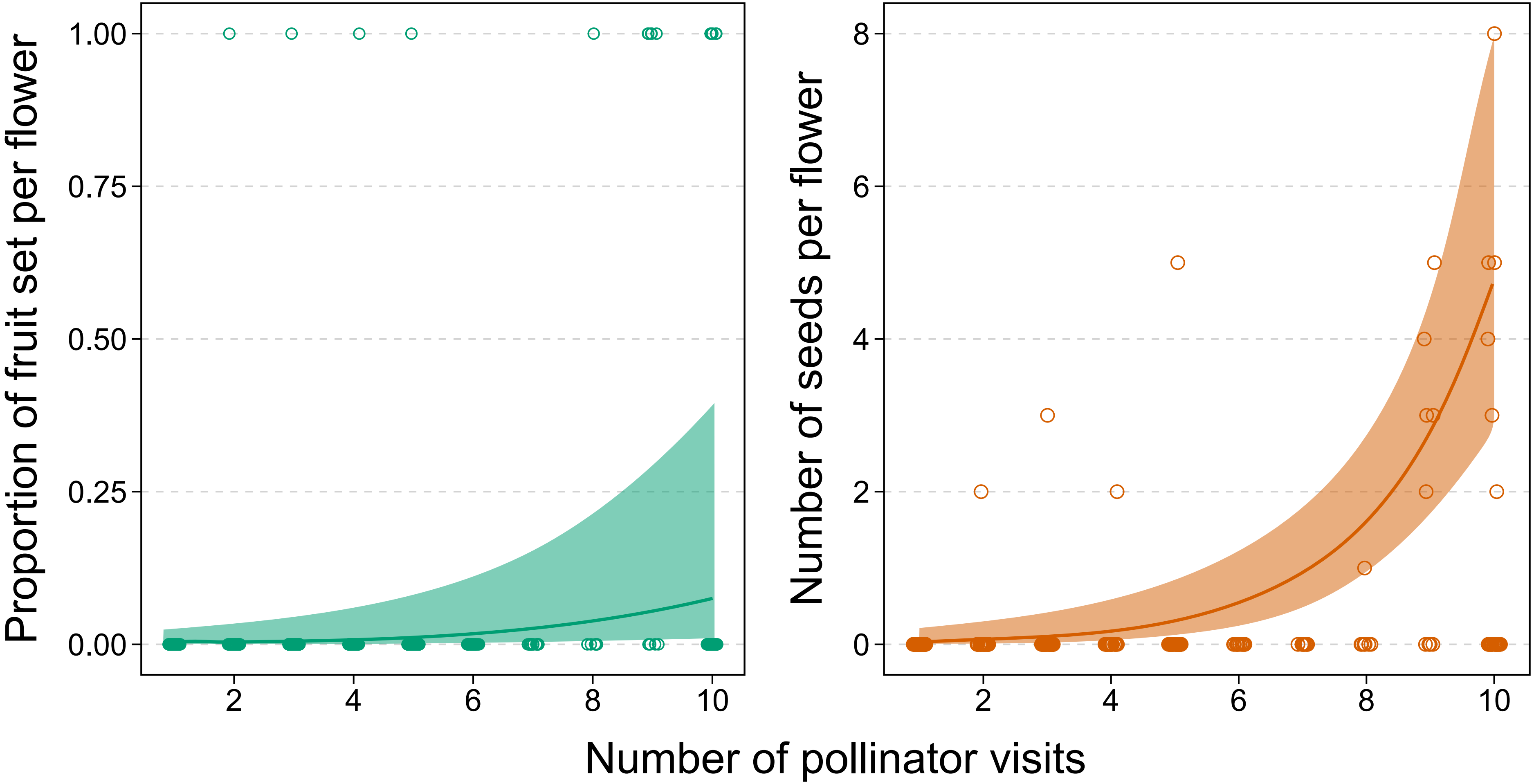

Figure S2. The proportion for flowers that set fruit and the number of seeds produced per flower with an increasing number of pollinator visits. Small semi-transparent circles represent the raw data for the presence or absence of a fruit, or the number of seeds produced per flower. Solid lines are model estimates and shaded ribbons are the model estimated 95% confidence intervals.

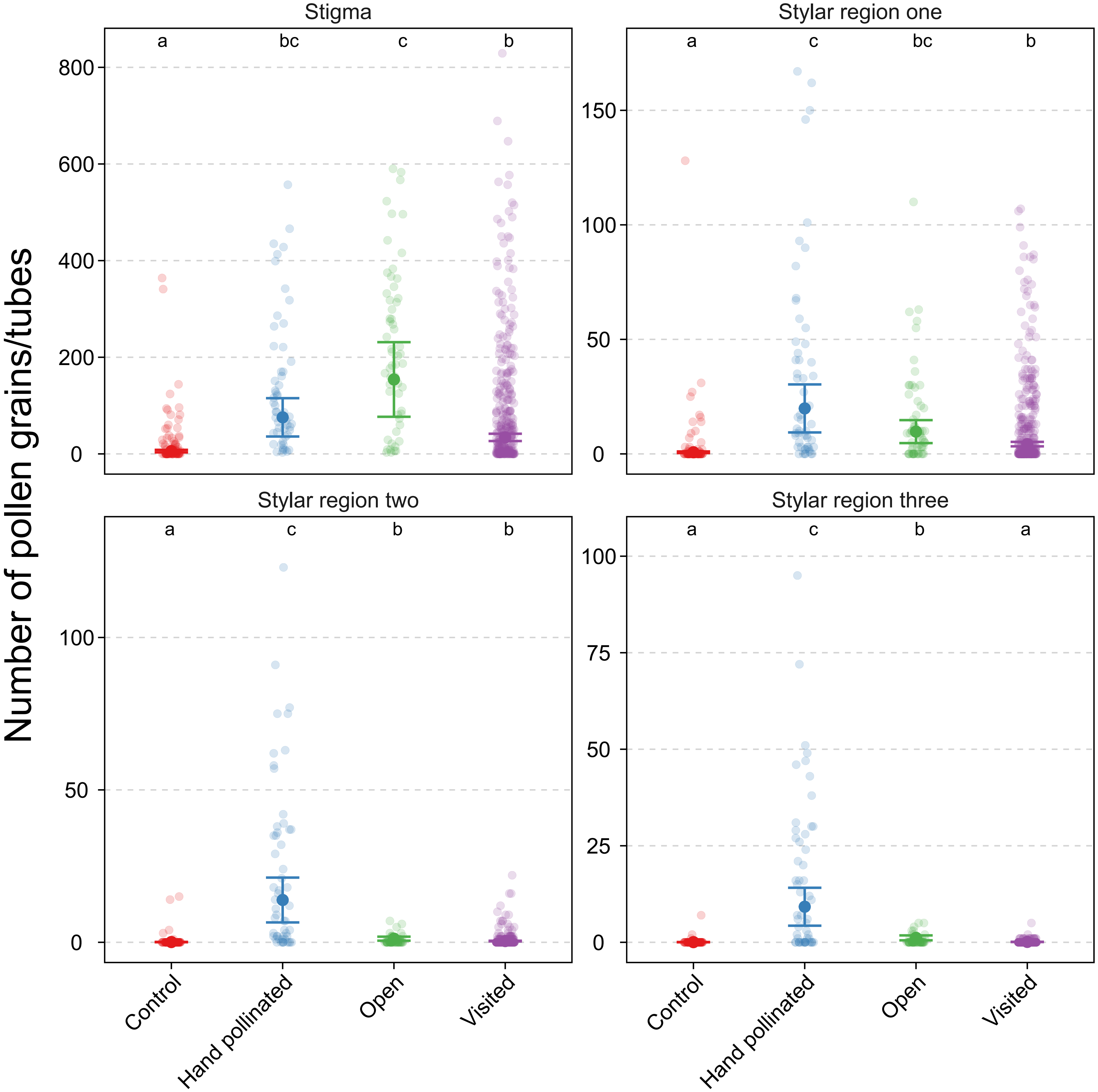

Figure S3. The number of pollen grains deposited on the stigma and the number of pollen tubes at the top, middle and bottom of styles for each pollination treatment (hand-pollinated: pollinated by hand with a compatible polliniser, open-pollinated: flowers left open to all pollinators until stigmas were no longer receptive, control: all pollinators excluded, visited: flowers visited one to twelve times by pollinators). Small semi-transparent circles are pollen grain or pollen tube counts from individual stigmas. Large solid circles are the model estimates for each treatment and error bars are the model estimated 95% confidence intervals. Letters above treatments denote statistically significant differences ($\alpha = 0.05$; FDR corrected) in the number of pollen grains or pollen tubes between treatments.

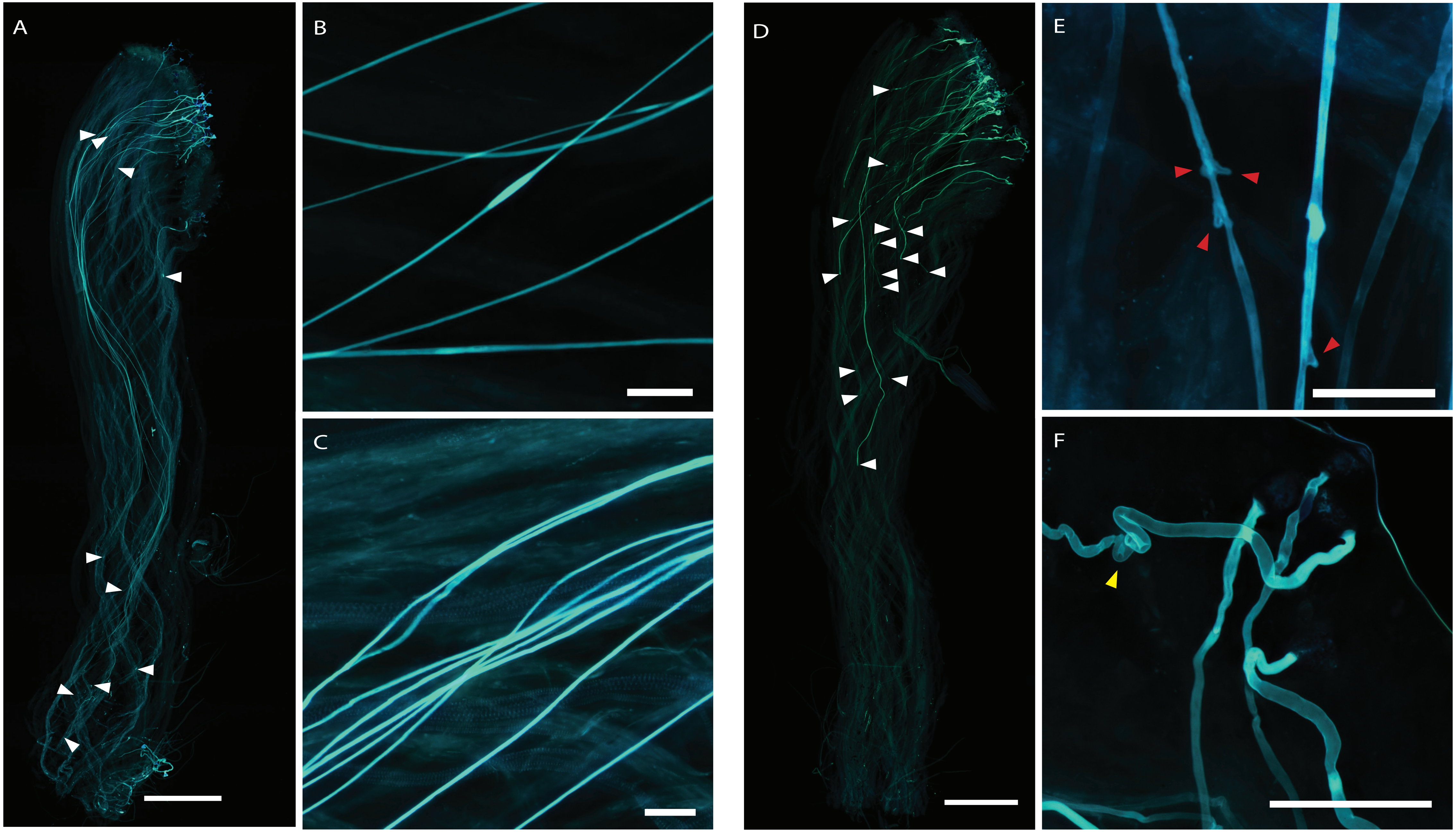

Figure S4. Pollen tubes in the style of "Pink Lady" apple. Panels A, B, and C show different styles from flowers that were cross-pollinated by hand with "Gala" apple pollen. Panels D, E, and F show styles that were visited 11, 7 and 5 times, respectively, by *Apis mellifera*. In panels A and D, white arrows mark maximum length of pollen tubes in the style. Pollen tubes arising from "Gala" pollen grow relatively straight (B, C), and frequently reach the bottom of the style (A), whereas pollen deposited by multiple visits from *A. mellifera* exhibited characteristics indicative of incompatibility (as in Jacquemart 2007; Oloumi and Rezanejhad 2009; Stott 1972), such as branching (E, marked with red arrows) and excessive twisting and bending (F, marked with a yellow arrow) and rarely grew past the middle region of the style (D). Scale bars in panels A and D are 1000 µm in length, scale bars in panels B, C, E, and F are 100 µm in length.
